# Opposing effects of clozapine and brexpiprazole on β-aminoisobutyric acid: Pathophysiology of antipsychotics-induced weight gain

**DOI:** 10.1038/s41537-023-00336-1

**Published:** 2023-02-08

**Authors:** Kouji Fukuyama, Eishi Motomura, Motohiro Okada

**Affiliations:** Department of Neuropsychiatry, Division of Neuroscience, Graduate School of Medicine, Mie University, Tsu, Mie 514-8507 Japan

**Keywords:** Schizophrenia, Synaptic transmission

## Abstract

Clozapine is one of the most effective antipsychotics and has the highest risk of weight gain and metabolic complications; however, the detailed pathophysiology of its clinical action and adverse reactions remains to be clarified. Therefore, the present study determined the chronic effects of clozapine (high risk of weight gain) and brexpiprazole (relatively low risk of weight gain) on intracellular and extracellular levels of β-aminoisobutyric acid (BAIBA) enantiomers, which are endogenous activators of AMP-activated protein kinase (AMPK). L-BAIBA is the dominant BAIBA enantiomer in the rat hypothalamus and cultured astrocytes, whereas L-BAIBA accounts for only approximately 5% of the total plasma BAIBA enantiomers. L-BAIBA displayed GABAB receptor agonistic action in the extracellular space and was released through activated astroglial hemichannels, whereas in the intracellular space, L-BAIBA activated AMPK signalling. Chronic administration of the effective doses of clozapine increased intracellular and extracellular levels of L-BAIBA in the hypothalamus and cultured astrocytes, whereas that of brexpiprazole decreased them. These results suggest that enhancing hypothalamic AMPK signalling by increasing intracellular L-BAIBA levels is, at least partially, involved in the pathophysiology of clozapine-induced weight gain and metabolic complications.

## Introduction

The life expectancy of patients with schizophrenia is 16 years shorter than the general population, with more than 30% of excess deaths attributable to metabolic complications^1^. Indeed, the prevalence of obesity in patients with psychiatric disorders (up to 60%) is twice that in the general population^2^. The mortality gap between patients with schizophrenia and the general population has been growing since atypical antipsychotics play important roles as an underlying risk-factor in metabolic complications in these groups^3,4^.

Adenosine-monophosphate (AMP)-activated protein kinase (AMPK) is an established target for treating insulin-resistant diabetes^5^. Indeed, the most widely used agent for the treatment of type-2 diabetes, metformin, activates AMPK^6^. Conversely, several antipsychotics, including clozapine and brexpiprazole, suppress AMPK signalling in the liver, thereby altering glucose metabolism^7,8^. A meta-analysis also demonstrated that metformin led to clinically meaningful prevention of clozapine-induced weight-gain^9^. AMPK controls the metabolism of individual cells in peripheral organs; however, hypothalamic AMPK seems to play fundamental roles in regulating both sides of the energy balances equation (feeding and energy expenditure) in whole-body^5^. Indeed, antipsychotics with a high-risk for weight gain (clozapine and olanzapine) enhance hypothalamic AMPK signalling, whereas lower-risk antipsychotics (lurasidone and brexpiprazole) suppress AMPK signalling^10–13^.

Antipsychotic-induced activation of hypothalamic AMPK is considered to be modulated by histamine H1 receptor inhbition^10,14^. Inhibition of the H1 receptor suppresses synthesis of inositol-trisphosphate (IP3), which enhances calcium-induced calcium release (CICR) via activating IP3 receptor^15,16^, leading to suppressed adenosine triphosphate (ATP) synthesis^3,17^. Therefore, H1 receptor inhibition likely activates hypothalamic AMPK via suppressed ATP synthesis^16^. This H1 receptor hypothesis is supported by the findings that clozapine activated and unaffected hypothalamic AMPK in wild-type and H1 receptor knockout mice, respectively^10^. However, like the H1 receptor, the 5-HT2A receptor also positively regulates IP3 synthesis^18^. Considering that most atypical antipsychotics are 5-HT2A receptor antagonists, it is important to explore how is 5-HT2A receptor blockade by brexpiprazole involved in AMPK signalling via decreased IP3 synthesis^3^.

Recently, β-aminoisobutyric acid (BAIBA) was re-discovered as a novel myokine that regulates adipose tissue browning, improves insulin sensitivity and protects against high-fat diet-induced obesity^19–21^. Interestingly, BAIBA increases the signalling of Akt, AMPK, and insulin receptor substrate and decreases the expression of gluconeogenic enzymes^21^. Based on these findings, BAIBA is considered a promising therapeutic target for metabolic disturbances. BAIBA is a GABA isomer composed of two enantiomers: D-BAIBA and L-BAIBA^22,23^. D-BAIBA is produced from thymine and metabolised by alanine-glyoxylate aminotransferase-2^24^, whereas L-BAIBA is produced from L-valine by 4-aminobutyrate aminotransferase (ABAT)^25–27^. ABAT is predominantly expressed in the brain and conversely degrades GABA^28–30^. Although we have already detected L-BAIBA release in the brain using microdialysis, its detailed release mechanism remains to be clarified^31^. Therefore, considering the metabolism and highly polar features of BAIBA, L-BAIBA is speculated to be the dominant enantiomer of BAIBA in the brain. However, functions of BAIBA in the brain have not been fully identified, except for binding to glycine and GABA_A_ receptors^32,33^.

Taken together with the previous findings that both clozapine (high-risk of weight gain) and brexpiprazole (relatively low-risk of weight gain) suppress AMPK signalling in the peripheral organs^7,8^, assuming that BAIBA enantiomers contribute to the pathophysiology of clinical actions and adverse reactionsof clozapine, clozapine (but not brexpiprazole) may enhance hypothalamic AMPK signalling via BAIBA enantiomer, leading to metabolic disorders. According to our hypothesis, this study determined the distribution of BAIBA enantiomers, their intracellular and extracellular functions, and their release mechanisms in the brain. Finally, the effects of chronic administration of clozapine and brexpiprazole on BAIBA enantiomers in the rat hypothalamus and astrocytes were determined.

## Materials and methods

### Experimental animals

All animal care and experimental procedures described in this report were performed according to the ethical guidelines established by the Institutional Animal Care and Use Committee at Mie University, Japan (No.2019-3, 24 May 2019) and are reported in accordance with the Animal Research: Reporting of In Vivo Experiments guidelines^34^. Male (6–7 weeks of age: *n* = 54) and neonatal (0–48 h of age: *n* = 18) Sprague-Dawley rats (SLC, Shizuoka, Japan) were used for in vivo and cultured astrocyte studies, respectively. Rats were housed individually in cages, kept in air-conditioned rooms (22 ± 2 °C) set to a 12 h light/dark cycle, and given free access to food/water.

### Chemical agents

Clozapine, brexpiprazole, carbenoxolone (CBX: hemichannel inhibitor), CGP52432 (GABA_B_ receptor antagonist), vigabatrin (ABAT inhibitor) and L-β-aminoisobutyric acid (L-BAIBA) were obtained from Funakoshi (Tokyo, Japan). All compounds were prepared on the day of the experiment. Clozapine was initially dissolved in 1 N HCl (50 mM)^35^. Brexpiprazole was initially dissolved in dimethyl sulfoxide at 25 mM^12^. The final dimethyl sulfoxide concentration was lower than 0.1% (v/v). CBX, CGP52432 and vigabatrin were directly dissolved in an experimental medium, such as a modified Ringer solution (MRS) (145 mM Na^+^, 2.7 mM K^+^, 1.2 mM Ca^2+^, 1.0 mM Mg^2+^, and 154.4 mM Cl^−^ adjusted to pH = 7.4 using 2 mM phosphate buffer and 1.1 mM Tris buffer), 100 mM K^+^ containing MRS (HKMRS) (47.7 mM Na^+^, 100 mM K^+^, 1.2 mM Ca^2+^ and 1.0 mM Mg^2+^), artificial cerebrospinal fluid (ACSF) (150 mM Na^+^, 3.0 mM K^+^, 1.4 mM Ca^2+^ and 0.8 mM Mg^2+^ and 5.5 mM glucose adjusted to pH = 7.3 using 20 mM HEPES buffer) or Dulbecco’s modified Eagle’s medium (D6546; Sigma-Aldrich, St. Louis, MO, USA) containing 10% foetal calf serum (fDMEM).

Effective doses of clozapine (5 mg/kg/day)^35,36^, brexpiprazole (10 mg/kg/day)^12,37^ and vigabatrin (75 mg/kg/day)^38^ were chronically administered to rats for 14-d using a subcutaneous osmotic pump (2ML_1; Alzet, Cupertino, CA, USA). Cultured astrocytes were chronically administrated either a therapeutic-relevant concentration of 0.3 μM brexpiprazole^12,37^ or 3 μM clozapine^37,39^ for 14-d. To inhibit ABAT and hemichannels, astrocytes were treated with 200 μM vigabatrin^40^ and 100 μM CBX^39^, respectively. In the microdialysis study, 50 μM CGP52432^41^ and 100 μM CBX^39^ dissolved in MRS or HKMRS were perfused in the dialysis probe.

### Microdialysis

All rats were weighed before the experiment. Rats were anaesthetised with 1.8% isoflurane and placed in a stereotaxic frame for 1 h. Concentric direct insertion dialysis probes were implanted in either the medial prefrontal cortex (A = +3.2 mm, *L* = +0.8 mm, V = −5.2 mm, relative to bregma; 0.22 mm diameter, 3 mm exposed membrane: EICOM, Kyoto, Japan) or hypothalamus (A = −3.2 mm, L = +0.5 mm, V = −9.2 mm, relative to bregma; 0.22 mm diameter, 1 mm exposed membrane: Eicom, Kyoto, Japan). Following surgery, rats were housed individually in cages during recovery and experiments and were provided food and water *ad libitum*.

Perfusion of MRS was started after 18 h of recovery from anaesthesia. Rats were placed into a system for freely moving animals (EICOM, Kyoto, Japan) equipped with a swivel (TCS2-23; ALS, Tokyo, Japan). The perfusion rate was set at 2 μL/min in all experiments using MRS or HKMRS. Dialysates were collected every 20 min. Extracellular levels were measured 8 h after starting perfusion. To determine the hypothalamic depolarisation-induced release of BAIBA enantiomers and GABA, the perfusion medium was switched from MRS containing with or without (control) 100 μM CBX^39^ to HKMRS containing the same agent for 20 min (HKMRS-evoked stimulation). To determine the effects of L-BAIBA on prefrontal extracellular dopamine level, the perfusion medium was switched from MRS containing with or without (control) 50 μM CGP52432^41^ to the same MRS containing 1 μM L-BAIBA for 120 min.

Dialysate was injected into ultra-high-performance liquid chromatography (UHPLC). After microdialysis, locations of dialysis probes were verified by histological examination using 200 μm thick brain tissue slices (Vibratome 1000; Technical Products, St. Louis, MO).

### Preparation of primary astrocyte culture

The preparation of cultured astrocytes was conducted mainly according to our previous studies^35,42^. Cultured astrocytes were prepared from cortical astrocytes of neonatal rats (0–48 h of age). Astrocytes were removed from flasks by trypsinisation and seeded directly onto a translucent polyethylene terephthalate membrane (1.0 μm) with 24-well plates (BD, Franklin Lakes, NJ) at a density of 100 cells/cm^2^ for experiments from the day after culturing for 14-d (DIV14) to DIV28. To determine the effects of chronic exposure to therapeutically relevant concentrations of clozapine, brexpiprazole and vigabatrin on astroglial BAIBA enantiomers and GABA, astrocytes were incubated in fDMEM containing clozapine (3 μM)^37,39^, brexpiprazole (0.3 μM)^43,44^ or vigabatrin (200 μM)^40^ for 14 d (DIV14-28). fDMEM was changed twice a week.

After washout by ACSF, cultured astrocytes were incubated in ACSF containing the same agent of chronic exposure to target agents, with or without 100 μM CBX, at 35 °C for 120 min in CO_2_ incubator. Astroglial hemichannels are not functional during the resting stage owing to their low opening probability^45^. Therefore, to determine activated hemichannel functions under physiological conditions, cultured astrocytes were electrically stimulated using ripple-burst evoked stimulation using busdrive amplifier (SEG-3104MG; Miyuki Giken, Tokyo, Japan). Sleep-spindle bursts are generally considered to be coupled with ripple bursts, as determined using wide-band electrocorticogram^45^. Recently, ripple burst has been shown to play important roles in sleep-dependent memory consolidation and other cognitive components^46^. Ripple-burst evoked stimulation was set at square-wave direct-current pulse output with a magnitude of 300 mV/mm^2^ ^45^. A set of ripple-burst evoked stimulations was composed of 10 stimuli at 200 Hz and 10 bursts (50% duty cycle) at burst intervals of 100 ms/s^45^. These ripple burst evoked stimulation patterns were regulated using LabChart version 8.2 software (AD Instruments, Dunedin, New Zealand).

### Extraction of cultured astrocytes, rat hypothalamus and plasma sample

After chronic administration of target agents for 14 d, the rat hypothalamus was dissected according to the method described by Glowinski and Iversen^47^. Both the hypothalamic tissue and cultured astrocytes were washed using ACSF. To apply the capillary immunoblotting system, after washout of hypothalamus tissue and cultured astrocytes were extracted using Minute Plasma Membrane Protein Isolation Kit (Invent Biotechnologies, Plymouth, MN, USA). Total protein levels were determined using a Protein Assay Reagent kit (FUJIFILM Wako Pure Chemical Corporation, Osaka, Japan).

To determine intracellular levels of BAIBA enantiomers, GABA, AMP, cAMP, ATP and IP3, cultured astrocytes or dissected rat hypothalamus were placed into 0.5 mL microtubes and homogenised with ultrasonic cell disrupter (VP-050N, Taitec, Koshigaya, Japan) using chilled 4 N perchloric acid with 4.3 mM EDTA^48^. The mixture was centrifuged at 10,000 *g* for 20 min at 4 °C. Filtered aliquots (5 μL) were injected into UHPLC or UHPLC equipped with mass spectrometry (UHPLC-MS).

### Determination of BAIBA enantiomer, GABA, and dopamine levels

BAIBA enantiomers and GABA were separated by UHPLC (xLC3185PU, Jasco, Tokyo, Japan) after dual derivatisation with isobutyryl-L-cysteine and o-phthalaldehyde^49^. Automated pre-column derivatives were prepared by drawing up 5 μL aliquot of the sample, standard, or blank solution and 5 μL of derivative reagent solution and allowing two to react in reaction vials for 5-min before injection. Derivatised samples (5 μL) were injected using autosampler (xLC3059AS; Jasco). The analytical column (Triat C18, particle size: 1.8 µm, 50 mm × 2.1 mm; YMC, Kyoto, Japan) was maintained at 35 °C, with flow rate set at 500 μL/min. A linear gradient elution programme was performed over 15 min with mobile phases A (0.1 M citrate buffer, pH = 3.5) and B (acetonitrile). The excitation/emission wavelength of the fluorescence detector (xLC3120FP, Jasco) was set to 345/455 nm.

Dopamine levels were determined by UHPLC (xLC3185PU; Jasco) and electrochemical detection (ECD-300; Eicom) with graphite carbon electrode set at +450 mV (vs Ag/AgCl reference electrode). The analytical column (Triart C18, particle size: 1.8 μm, 30 × 2.1 mm; YMC) was maintained at 25 °C, and the flow rate of the mobile phase was set at 500 μL/min. The mobile phase was composed of 0.1 M acetate buffer containing 1% methanol and 50 mg/L EDTA-2Na (pH = 6.0).

### Determination of intracellular levels of cAMP, AMP, ATP and IP3

AMP, cAMP, ATP and IP3 levels were analysed by UHPLC-MS (Acquity UHPLC H-Class equipped with Acquity SQ detector; Waters, Milford, MA). Samples (5 μL aliquots) were automatically injected by autosampler (Acquity UHPLC Sample Manager FTN; Waters) and separated by graphite carbon column (particle size: 3 μm, 150 × 2.1 mm; Hypercarb, Thermo) maintained at 450 μL/min at 40 °C.

The UHPLC-MS procedure for determining levels of cAMP, AMP and ATP were as follows. A linear gradient elution programme was used for over 10 min with mobile phases A (1 mM ammonium acetate buffer, pH = 11) and B (100% acetonitrile). Nitrogen flow rates of desolvation and cone were set at 750 and 5 L/h, respectively. The desolvation temperature was set to 450 °C. Cone voltages for the determination of cAMP (m/z = 330.3), AMP (m/z = 348.2), and ATP (m/z = 508.2) were 42, 40, and 34 V, respectively.

The UHPLC-MS procedure for the determination of IP3 level was as follows. A linear gradient elution programme was used for over 10 min with mobile phases A (10% acetate) and B (100% acetonitrile). The nitrogen flow rates of desolvation and cone were set at 750 and 5 L/h, respectively. The desolvation temperature was set to 450 °C. The cone voltage used to determine IP3 (m/z = 421.1) was 35 V.

### Capillary immunoblotting analysis

Capillary immunoblotting analysis was performed using Wes (ProteinSimple, Santa Clara, CA, USA) according to the manufacturer’s instructions. Lysates mixed with the master mix (ProteinSimple) were heated at 95 °C for 5 min. Samples, blocking reagents, primary antibodies, horseradish peroxidase (HRP)-conjugated secondary antibodies, chemiluminescent substrate (SuperSignal West Femto; Thermo Fisher Scientific, Waltham, MA, USA), and separation and stacking matrices were dispensed into designated wells of a 25-well plate. After plate loading, separation electrophoresis and immunodetection were performed in fully automated capillary system. Capillaries were incubated with blocking reagent for 15 min, and target proteins were probed with primary antibodies, followed by incubation with HRP-conjugated secondary antibodies (Anti-Rabbit IgG HRP, A00098, 10 μg/mL; GenScript, Piscataway, NJ, USA). Antibodies against AMPKα (2603, 1:50; Cell Signalling, Danvers, MA, USA), phosphorylated AMPKα (2535, 1:50; Cell Signalling) and GAPDH (NB300-322, 1:100, Novus Biologicals, Littleton, CO, USA) were diluted in an antibody diluent (Immuno Shot Platinum, CosmoBio)^50^.

### Data analysis

All experiments were designed with groups containing equal number of animals (*n* = 6) without formal power analysis, in accordance with previous studies^12,35,37,39–41,44,51,52^. All values are expressed as mean±standard deviation (SD), and *p* < 0.05 (two-tailed) was considered statistically significant for all tests. Drug levels for chronic administration were selected based on values reported in previous studies^12,35–38^. Where possible, we aimed to randomise and blind the data. To determine levels of BAIBA enantiomers, GABA, cAMP, IP3, AMP, ATP and protein expression, the sample order on autosamplers and Wes was selected using random number tables.

Effects of chronic administration of clozapine, brexpiprazole and vigabatrin on levels of BAIBA enantiomers, GABA and AMPK were analysed via analysis of variance (ANOVA) with Tukey’s post hoc test or Student T-test using BellCurve for Excel version 3.2 (Social Survey Research Information Co. Ltd., Tokyo, Japan). Effects of chronic administration of clozapine and brexpiprazole on intracellular levels of cAMP, AMP, ATP and IP3 were analysed using Student’s t-test (BellCurve for Excel). Effects of chronic administration of clozapine, brexpiprazole and vigabatrin on extracellular levels of BAIBA enantiomers and GABA were assessed using multivariate analysis of variance (MANOVA) with Tukey’s post-hoc test (BellCurve for Excel). Interaction between perfusion with L-BAIBA and CGP52432 on extracellular dopamine levels in the prefrontal cortex was also analysed using MANOVA with Tukey’s post-hoc test. When the data did not violate the assumption of sphericity (*p* > 0.05), the F-value of MANOVA was analysed using sphericity-assumed degrees of freedom. However, if the assumption of sphericity was violated (*p* < 0.05), F-value was analysed using chi-Muller’s corrected degrees of freedom. When the F-value for the level/time factors of the MANOVA was significant, the data were analysed using Tukey’s post-hoc test. The rat body weight between before and after chronic administration of vehicle, clozapine (5 mg/kg/day) and brexpiprazole (10 mg/kg/day) using a subcutaneous osmotic pump were analysed by one-way analysis of variance (ANOVA) with Tukey’s post hoc test.

The data and statistical analyses complied with the recommendations of the British Journal of Pharmacology on experimental design and analysis in pharmacology^53^.

## Results

### Body weight

The rats’ body weights before implantation of the subcutaneous osmotic pump and after experiments were determined. Body weight of administration of vehicle (control), clozapine (5 mg/kg/day) and brexpiprazole (10 mg/kg/day) were 190.7 ± 11.4 g to 260.5 ± 18.6 g, 183.0 ± 11.7 g to 271.4 ± 26.6 g and 188.6 ± 10.6 g to 262.8 ± 12.1 g, respectively. Therefore, chronic clozapine administration increased body weight (88.4 ± 17.1 g: *p* < 0.05), but brexpiprazole did not affect (74.1 ± 5.6 g) compared to control (69.9 ± 8.6 g) [F(2,17) = 4.27(*p* < 0.05)].

### BAIBA enantiomer levels in plasma, hypothalamus and astrocytes

**Fig. 1:**
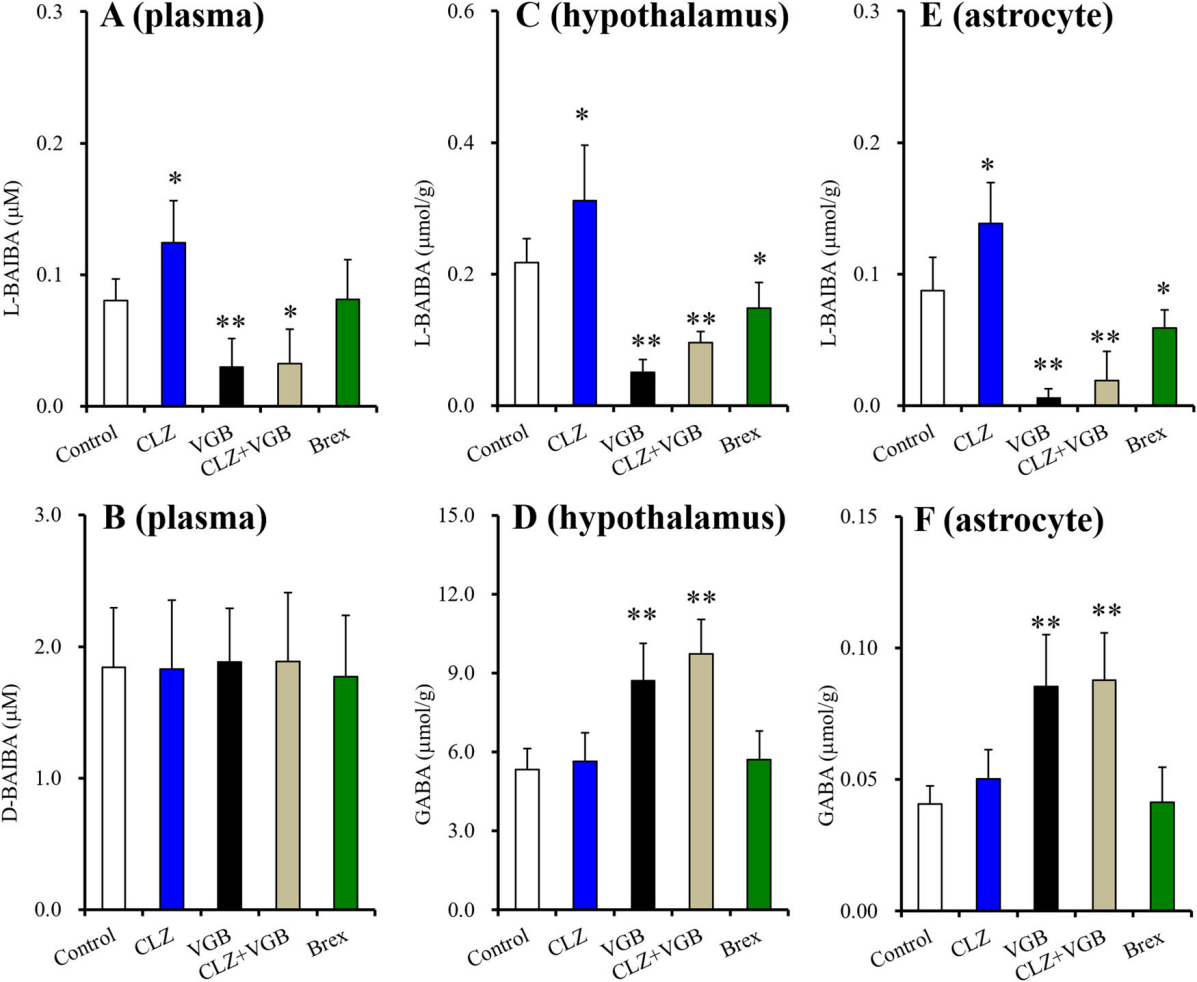
Effects of systemically chronic administration of effective doses of clozapine, brexpiprazole and vigabatrin on plasma concentrations of BAIBA enantiomers. Effects of systemically chronic administration (for 14-d) of effective doses of clozapine (CLZ: 5 mg/kg/day), brexpiprazole (Brex: 10 mg/kg/day) and vigabatrin (75 mg/kg/day) on plasma concentrations of BAIBA enantiomers, L-BAIBA (**A**) and D-BAIBA (**B**). Effects of systemically chronic administration (for 14d) of effective doses of clozapine (CLZ: 5 mg/kg/day), brexpiprazole (Brex: 10 mg/kg/day) and vigabatrin (75 mg/kg/day) on levels of L-BAIBA (C) and GABA (**D**) in the rat hypothalamus. Effects of chronic exposure (for 14d) to therapeutic relevant levels of clozapine (CLZ: 3 μM), brexpiprazole (Brex: 0.3 μM) and vigabatrin (200 μM) on levels of L-BAIBA (**E**) and GABA (**F**) in the cultured astrocytes. Ordinate: mean ± SD (*n* = 6) of plasma concentration of BAIBA enantiomer (μM). *p < 0.05, **p < 0.01: relative to control by ANOVA with Tukey’s post-hoc test or student T-test.

Initial plasma levels of D-BAIBA and L-BAIBA were 1.84 ± 0.45 μM and 0.08 ± 0.02 μM, respectively (Fig. 1a, b). In the literature, the plasma levels of D-BAIBA and L-BAIBA have been inconsistent^54–56^. The present results closely resemble the plasma levels reported previously by Stautemas et al. ^56^. Therefore, D-BAIBA is a major BAIBA enantiomer (>95%) in the blood (peripheral organs). The plasma D-BAIBA level was not affected by chronic administration of clozapine (5 mg/kg/day), brexpiprazole (10 mg/kg/d) or vigabatrin (400 mg/kg/d)(Fig. 1b). Chronic brexpiprazole administration did not affect plasma L-BAIBA level. In contrast, chronic administrations of clozapine and vigabatrin increased and decreased plasma L-BAIBA levels, respectively (Fig. 1a). Under the ABAT inhibition by vigabatrin, clozapine did not increase plasma L-BAIBA level [F(3,20) = 19.7(*p* < 0.01)] (Fig. 1a).

In the hypothalamus, the L-BAIBA level was 0.22 ± 0.04 μmol/g protein, but D-BAIBA was not detectable (Fig. 1c). Therefore, contrary to peripheral organs, L-BAIBA is the dominant BAIBA enantiomer in the hypothalamus. The hypothalamic GABA level was determined since L-BAIBA and GABA are synthesised and degraded by ABAT, respectively^30,57^. Indeed, chronic vigabatrin administration decreased and increased the hypothalamic levels of L-BAIBA and GABA, respectively (Fig. 1c, d). Chronic brexpiprazole administration decreased L-BAIBA levels but did not affect GABA levels (Fig. 1c, d). Chronic clozapine administration increased the L-BAIBA level without affecting GABA level; however, under ABAT inhibition by vigabatrin, clozapine did not affect levels of L-BAIBA [F(3,20) = 36.5 (*p* < 0.01)] or GABA [F(3,20) = 20.7(*p* < 0.01)] (Fig. 1c, d).

In the cultured astrocytes, levels of L-BAIBA and GABA were 0.09 ± 0.03 and 0.04 ± 0.01 μmol/g protein, respectively (Fig. 1e, f). Chronic brexpiprazole administration decreased astroglial L-BAIBA levels but unaffected GABA levels (Fig. 1e, f). Chronic vigabatrin administration decreased and increased levels of L-BAIBA and GABA, respectively (Fig. 1e, f). Chronic clozapine administration increased L-BAIBA level, whereas under ABAT inhibition by vigabatrin, levels of L-BAIBA were unaffected by clozapine [F(3,20) = 43.4 (*p* < 0.01)](Fig. 1e, f). Moreover, chronic clozapine administration did not affect GABA levels, even under ABAT inhibition [F(3,20) = 15.8(*p* < 0.01)](Fig. 1e, f). Therefore, the metabolism of L-BAIBA in astrocytes conceivably resembled that in the hypothalamus since the effects of chronic administration of clozapine, vigabatrin and brexpiprazole on astroglial intracellular levels of L-BAIBA and GABA showed similar tendencies.

### Effects of clozapine, brexpiprazole and L-BAIBA on AMPK signalling

#### Effects of chronic administration of clozapine and brexpiprazole on second messengers in the cultured astrocytes and hypothalamus

**Fig. 2:**
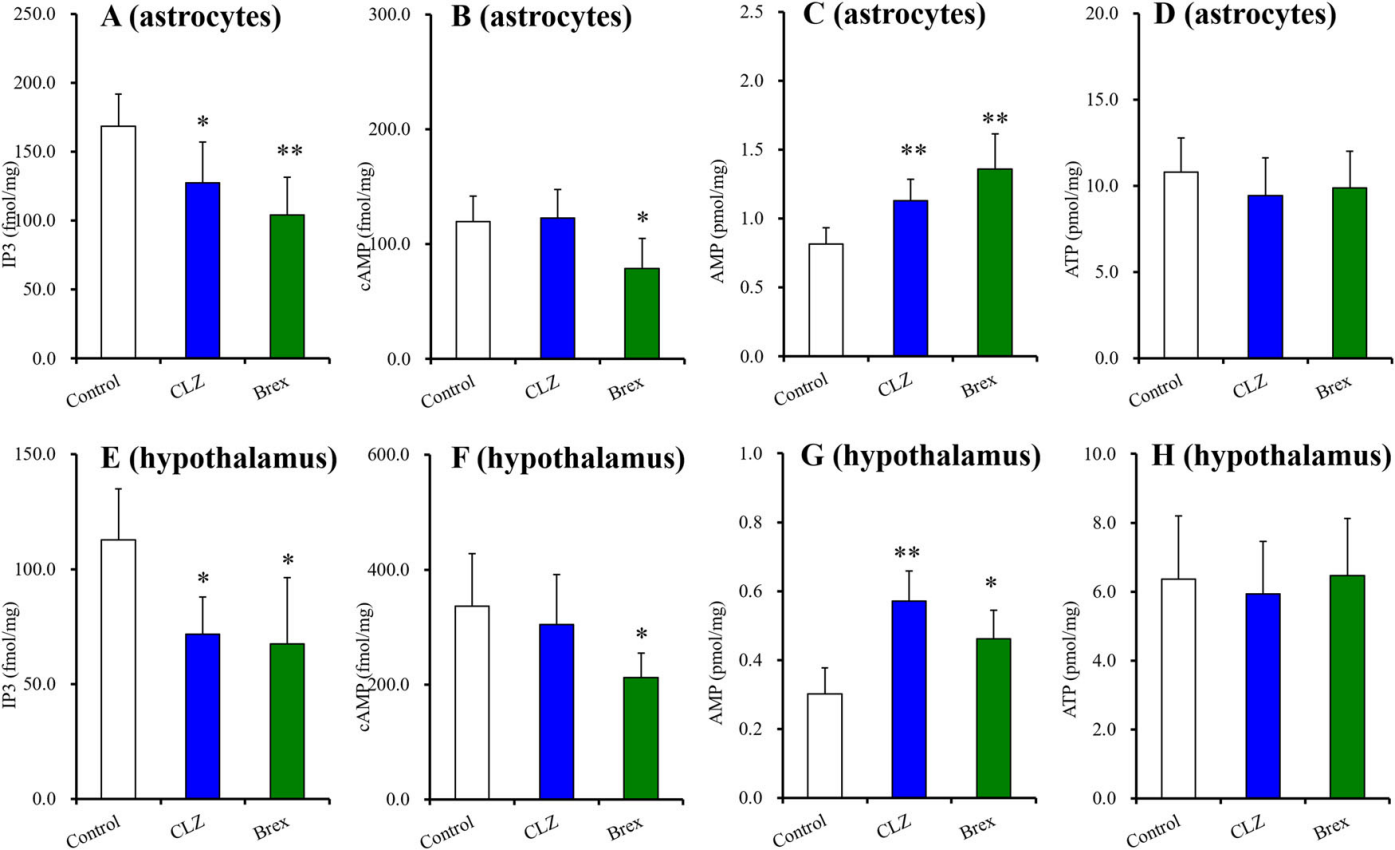
Effects of chronic exposure to therapeutic relevant concentration of clozapine and brexpiprazole (0.3 μM) on levels of IP3, cAMP, AMP and ATP in the cultured astrocytes and the hypothalamus. Effects of chronic exposure (for 14-d) to therapeutic relevant concentration of clozapine (3 μM) and brexpiprazole (0.3 μM) on levels of IP3 (**A**), cAMP (**B**), AMP (**C**) and ATP (**D**) in the cultured astrocytes. Effects of chronic administration (for 14-d) of effective dose of clozapine (5 mg/kg/day) and brexpiprazole (10 mg/kg/day) on levels of IP3 (**E**), cAMP (**F**), AMP (**G**) and ATP (**H**) in the hypothalamus. Ordinate: mean ± SD (*n* = 6) of intracellular levels of IP3 and cAMP (fmol/mg protein), or AMP and ATP (pmol/mg). *p < 0.05, **p < 0.01: relative to control (agent free) by student T-test.

Chronic administrations of clozapine and brexpiprazole decreased and increased intracellular levels of IP3 and AMP, respectively, without affecting ATP levels in both cultured astrocytes and hypothalamus (Fig. 2). Decreased IP3 and increased AMP by chronic clozapine administration reasonably support a candidate mechanism of clozapine-induced activation of AMPK signalling due to suppressing IP3 production; however, H1 receptor hypothesis cannot explain the mechanisms of brexpiprazole-induced inhibition of AMPK signalling. The intracellular cAMP level was decreased by brexpiprazole but remained unaffected by clozapine in both the cultured astrocytes and hypothalamus (Fig. 2). It has been established that cAMP enhances and inhibits AMPK signalling via activation of EPAC and PKA activities, respectively^58^. We have already reported that the decreased cAMP levels by brexpiprazole possibly contributed to the suppression of AMPK signalling^43^, whereas the detailed mechanisms of our speculation remained to be determined.

#### Effects of chronic administration of clozapine and L-BAIBA on AMPK signalling in cultured astrocytes

Previous studies revealed that systemic administration of clozapine and brexpiprazole activated and suppressed AMPK signalling^43,59^. Peripherally systemic administration of L-BAIBA cannot be used to examine its effect on AMPK activity in the brain since L-BAIBA has difficulty penetrating the blood-brain barrier due to its high polarity, similar to GABA. Therefore, in this study, we investigated the effects of chronic exposure to L-BAIBA on AMPK signalling in cultured astrocytes.

**Fig. 3:**
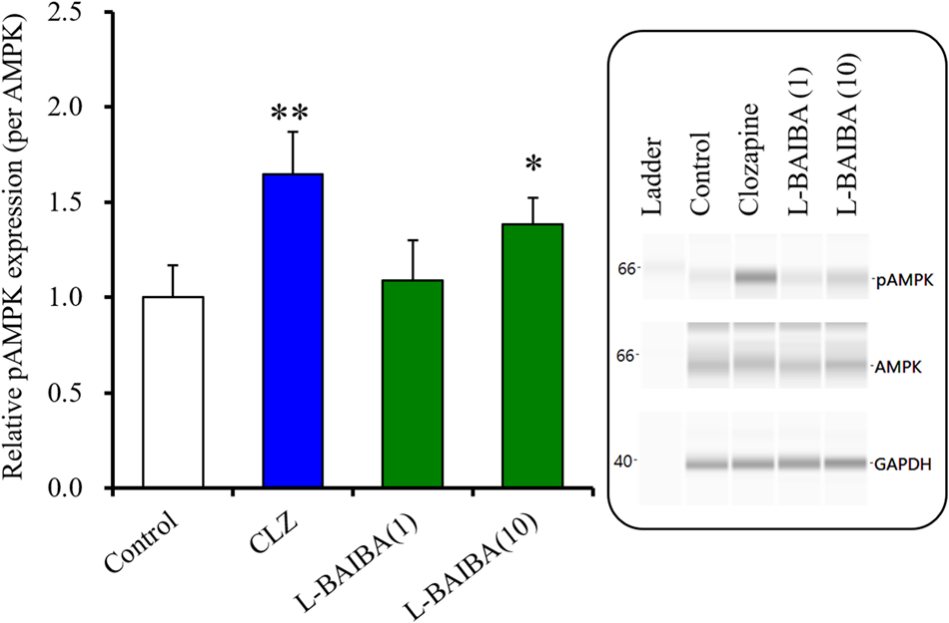
Effects of chronic exposure (for 14-d) to therapeutic relevant concentration of clozapine (3 μM) and L-BAIBA (1 μM and 10 μM) on phosphorylated AMPK (pAMPA) of cultured astrocytes. In left side histograms, ordinate: mean ± SD (*n* = 6) of the relative levels of pAMPK. *p < 0.05, **p < 0.01: relative to control (drug free) by one-way ANOVA with Tukey’s post hoc test. Right side panels indicate their pseudo-gel images of capillary immunoblotting.

Chronic exposure to a therapeutically relevant concentration of clozapine (3 μM)^37,39^ enhanced astroglial AMPK signalling (Fig. 3), resembling effects of systemic clozapine administration^60^. Cultured astrocytes were chronically administered by L-BAIBA (1 μM), an amount equivalent to intracellular L-BAIBA level in the hypothalamus; however, 1 μM L-BAIBA did not affect AMPK signalling (Fig. 3). Contrary, 10 μM L-BAIBA enhanced AMPK signalling in the astrocytes (Fig. 3). This result suggests that effective level of extracellular L-BAIBA (10 μM) is comparable to that at which L-BAIBA enhanced AMPK signalling in hepatocytes (5 μM) and muscle (10 μM)^20,21^.

### Extracellular levels and functions of L-BAIBA

#### Extracellular levels of L-BAIBA and GABA in the hypothalamus

Extracellular functions of BAIBA in the brain have been identified as agonists of glycine and GABA_A_ receptors^32,33^. Additionally, we have already detected L-BAIBA release in the brain using microdialysis, but its release mechanism remains to be clarified^31^. When L-BAIBA is released to extracellular space via a specific regulation mechanism, L-BAIBA is possibly a candidate transmitter or modulator in the brain.

**Fig. 4:**
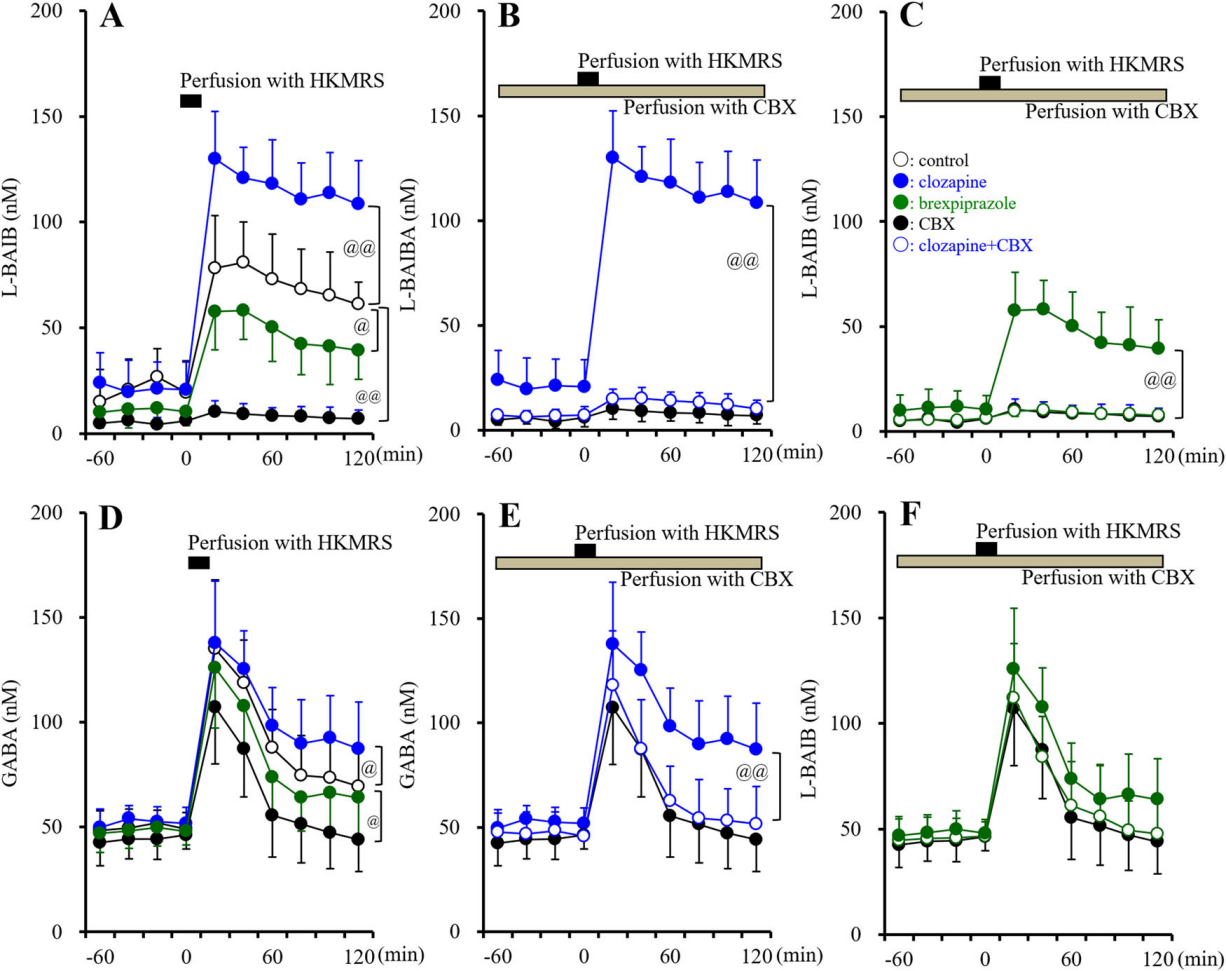
Effects of systemically chronic administration of effective doses of clozapine, brexpiprazole and local administration of non-selective astroglial hemichannel inhibitor, carbenoxolone on extracellular levels of L-BAIBA and GABA in the rat hypothalamus using microdialysis. Effects of systemically chronic administration (for 14-d) of effective doses of clozapine (CLZ: 5 mg/kg/day), brexpiprazole (Brex: 10 mg/kg/day) and local administration of non-selective astroglial hemichannel inhibitor, carbenoxolone (CBX: 100 μM) on extracellular levels of L-BAIBA (**A**–**C**) and GABA (**D**–**F**) in the rat hypothalamus using microdialysis. Ordinate: mean ± SD (*n* = 6) of extracellular levels of L-BAIBA and GABA in the hypothalamus (nM). Abscissa: time after HKMRS-evoked stimulation (100 mM K^+^ containing perfusion medium) (min). Black and grey bars indicated the perfusion with HKMRS for 20 min and perfusion with 100 μM CBX containing perfusate, respectively. @*p* < 0.05, @@p < 0.01: relative to control (**A**, **D**) or clozapine alone (**B**, **E**) or brexpiprazole anone (**C**, **F**) by MANOVA with Tukey’s post hoc test. Perfusion with 100 μM CBX inhibited HKMRS-evoked releases of L-BAIBA [F_time_(2.8,28.4) = 54.7(*p* < 0.01), F_CBX_(1,10) = 56.1(*p* < 0.01), F_time*clozapine_(2.8,28.4) = 41.2(*p* < 0.01)] and GABA [F_time_(2.4,24.1) = 116.2(*p* < 0.01), F_CBX_(1,10) = 5.4(*p* < 0.05), F_time*clozapine_(2.4,24.1) = 3.2(*p* < 0.01)]. Chronic administration of clozapine increased HKMRS-evoked releases of L-BAIBA [F_time_(6,60) = 224.4(*p* < 0.01), F_clozapine_(1,10) = 14.9(*p* < 0.01), F_time*clozapine_(6,60) = 2.1(*p* > 0.05)] and GABA [F_time_(6,60) = 114.7 (*p* < 0.01), F_clozapine_(1,10) = 11.1(*p* < 0.01), F_time*clozapine_(6,60) = 7.45(*p* < 0.05)], which were suppressed by hemichannel inhibitor, CBX. Chronic administration of brexpiprazole decreased HKMRS-evoked release of L-BAIBA [F_time_(6,60) = 97.4 (*p* < 0.01), F_brexpiprazole_(1,10) = 5.2(*p* < 0.05), F_time*brexpiprazole_ (6,60) = 0.6(*p* > 0.05)] but did not affect GABA release.

Basal extracellular levels of L-BAIBA (20.5 ± 14.2 nM) and GABA (48.5 ± 9.2 nM) were detectable in the hypothalamus using microdialysis (Fig. 4). HKMRS-evoked stimulation increased extracellular levels of L-BAIBA and GABA, while extracellular D-BAIBA level remained undetectable. Increasing patterns of HKMRS-evoked release of L-BAIBA and GABA were not identical. GABA release was increased by HKMRS-evoked stimulation but gradually decreased thereafter (Fig. 4d). In contrast, L-BAIBA level was also increased by HKMRS-evoked stimulation, but the subsequent gradual decreasing phase was not observed (Fig. 4a). Perfusion with 100 μM CBX (hemichannel inhibitor) inhibited HKMRS-evoked releases of both L-BAIBA and GABA (Fig. 4a, d). CBX inhibited predominantly HKMRS-evoked L-BAIBA release rather than that of GABA (Fig. 4a, d). Significantly, the CBX did not affect the initial phase of HKMRS-evoked GABA release, whereas the late gradual decreasing phase of HKMRS-evoked GABA release was decreased by CBX (Fig. 4d). These discrepancies suggest that inactivation in the extracellular space or release system of L-BAIBA is different from that of GABA.

Chronic administration of clozapine and brexpiprazole increased and decreased HKMRS-evoked releases of L-BAIBA, respectively (Fig. 4a–c). During the perfusion with 100 μM CBX (hemichannel inhibitor), the stimulatory effect of clozapine and the inhibitory effect of brexpiprazole on HKMRS-evoked L-BAIBA release was not observed (Fig. 4b, c). Chronic administration of clozapine weakly increased HKMRS-evoked GABA release, whereas chronic brexpiprazole administration did not affect (Fig. 4d). Perfusion with 100 μM CBX prevented the stimulatory effect of clozapine on HKMRS-evoked GABA release, but brexpiprazole was not affected by CBX (Fig. 4e, f). Therefore, these results suggest that clozapine and brexpiprazole probably increased and decreased astroglial L-BAIBA release, respectively, at least partially, through activated astroglial hemichannels.

#### Astroglial L-BAIBA release

Microdialysis study suggested that L-BAIBA is a candidate gliotransmitter, which is released via activated astroglial hemichannel, whereas HKMRS-evoked stimulation (100 mM K^+^) is toxic and not physiological stimulation^15,61,62^. To confirm whether the astroglial release of L-BAIBA receives regulation of the gliotransmitter releasing system, basal and ripple-evoked astroglial L-BAIBA release was determined^45^.

**Fig. 5:**
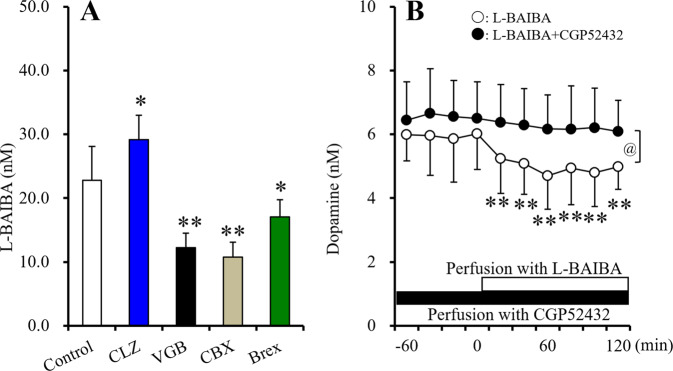
Effects of chronic exposure to therapeutic relevant levels of clozapine, brexpiprazole, vigabatrin (200 μM) and acute exposure to carbenoxolone on astroglial ripple-evoked releases of L-BAIBA, and interaction between L-BAIBA and CGP52432 (GABAB receptor antagonist) on dopamine release using microdialysis. Effects of chronic exposure (for 14-d) to therapeutic relevant levels of clozapine (CLZ: 3 μM), brexpiprazole (Brex: 0.3 μM), vigabatrin (200 μM) and acute exposure to CBX (100 μM) on astroglial ripple-evoked releases of L-BAIBA (**A**). Ordinate: mean ± SD (*n* = 6) of levels of L-BAIBA (nM). *p < 0.05, **p < 0.01: relative to control by student T-test. Ripple-evoked releases of were detectable. Interaction between perfusion with L-BAIBA (1 μM) and selective GABAB receptor antagonist (CGP52432: 50 μM) on extracellular dopamine level in the prefrontal cortex using microdialysis (**B**). Ordinate: mean ± SD (*n* = 6) of extracellular dopamine level in the prefrontal cortex (nM). Abscissa: time after perfusion with L-BAIBA containing MRS. White and black bars indicated the perfusion with L-BAIBA and CGP52432, respectively. **p < 0.01: relative to pre-perfusion period of L-BAIBA, and @*p* < 0.05: relative to perfusion with L-BAIBA plus CGP52432 by MANOVA with Tukey’s post-hoc test. L-BAIBA decreased extracellular dopamine level, but the inhibitory effects of L-BAIBA was prevented by CGP52432 [Ftime(6,60) = 8.9 (*p* < 0.01), FCGP(1,10) = 7.1(*p* < 0.05), Ftime*CGP(6,60) = 7.45(*p* < 0.05)].

During the resting stage, astroglial releases of D-BAIBA, L-BAIBA and GABA were not detectable. However, ripple-evoked stimulation invoked astroglial L-BAIBA release, which CBX and vigabatrin inhibited (Fig. 5a). Contrary to L-BAIBA, ripple-evoked astroglial releases of D-BAIBA and GABA were not detected. Chronic clozapine administration increased ripple-evoked L-BAIBA release, but brexpiprazole weakly decreased. Both in vivo microdialysis and in vitro cultured astrocyte studies indicate that L-BAIBA is released from astrocytes through a non-exocytotic process, such as activated astroglial hemichannel. Thus, L-BAIBA is a candidate gliotransmitter.

#### Effects of local administration of L-BAIBA on dopamine release in the rat prefrontal cortex

It has been previously reported that BAIBA activates GABA_A_ receptor^33^; however, the effects of L-BAIBA on GABA_B_ receptors have not been clarified. Considering the similar molecular structure and GABA_A_ receptor agonistic action of BAIBA, the possibility that L-BAIBA affects GABA_B_ receptors is a scientific issue that should be pharmacologically confirmed. Perfusion with the GABA_B_ receptor agonists SKF97541 and baclofen decreases extracellular dopamine levels in the prefrontal cortex, but the GABA_B_ receptor antagonist CGP52432 inhibits these reductions in dopamine release at 50 μM^33,41^. According to the previous findings, to clarify the effects of L-BAIBA on GABA_B_ receptors based on these previous findings, results of perfusion with 1 μM L-BAIBA and CGP52432 on dopamine release in the prefrontal cortex were determined using microdialysis. Perfusion with 1 μM L-BAIBA decreased extracellular dopamine level, similar to baclofen^33^, but the inhibitory effects of perfusion with 1 μM L-BAIBA were prevented by perfusion with 50 μM CGP52432 (Fig. 5b). Therefore, these results suggest that L-BAIBA acts as GABA_B_ receptor agonist.

## Discussion

### L-BAIBA is a candidate GABAergic gliotransmitter

The present study demonstrated that L-BAIBA is the dominant BAIBA enantiomer in the hypothalamus and astrocytes, whereas, in the blood, D-BAIBA is the major BAIBA enantiomer. Intracellular metabolic kinetics of L-BAIBA was opposite to that of GABA via ABAT since L-BAIBA and GABA are synthesised and depredated by ABAT, respectively^27^. Specifically, inhibition of ABAT by vigabatrin increased and decreased intracellular levels of GABA and L-BAIBA, respectively. Additionally, the present study also identified that L-BAIBA likely enhances GABA_B_ receptors. Together, with its similar molecular structure to GABA and GABA receptor agonistic action of L-BAIBA^21,33^, L-BAIBA is a candidate endogenous GABAergic modulator. However, the release process of L-BAIBA differs from that of GABA. Our microdialysis study detected lower hypothalamic basal L-BAIBA levels than GABA, whereas HKMRS-evoked stimulation (100 mM K^+^) for 20 min fully activated both hypothalamic releases of GABA and L-BAIBA. Temporal inactivation patterns between extracellular GABA and L-BAIBA were quite different as GABA showed an eventual gradual decrease after HKMRS-evoked stimulation, but persistent increasing L-BAIBA were observed for more than 120 min. Even considering the degradation of GABA in extracellular space by ABAT, these discrepancies in extracellular kinetics between GABA and L-BAIBA suggest the existence of different extracellular inactivation systems or releasing systems of L-BAIBA against the GABAergic system. In this study, despite the almost equal intracellular levels of L-BAIBA and GABA in astrocytes, a greater proportion of L-BAIBA was released through activated astroglial hemichannels rather than through exocytosis, whereas GABA was mainly released via neuronal exocytosis^63^. Therefore, L-BAIBA is a candidate endogenous GABAergic gliotransmitter in extracellular space.

Chronic administration of clozapine and brexpiprazole weakly increased and unaffected protein expression of connexin43 in the plasma membrane, respectively^12,37,39^. These previous findings can explain mechanisms by which chronic administration of clozapine and brexpiprazole increased and decreased L-BAIBA releases, respectively. Indeed, hemichannel blockade drastically inhibited astroglial L-BAIBA release. Additionally, chronic administration of clozapine and brexpiprazole increased and decreased intracellular L-BAIBA levels, respectively. Based on these results, it can be interpreted that CLZ increases astroglial L-BAIBA release via increasing both intracellular concentration as a source and hemichannels as a player. However, the two findings suggest that ABAT activity may not be the primary mechanism of stimulatory effects of clozapine on astroglial L-BAIBA release. First, the optimum pH of ABAT is approximately 9.0^27^. The others in this study, neither clozapine nor brexpiprazole, affected intracellular levels of GABA, which is a counter substance to L-BAIBA for ABAT. Therefore, upstream of ABAT in the L-BAIBA metabolic pathway is potentially affected by clozapine and brexpiprazole. We shall report the detailed mechanisms underlying the discrepant effects of clozapine and brexpiprazole on L-BAIBA metabolism in future studies.

Recently, X-ray crystal structure analysis of GABA_B_ receptors suggested that clozapine possibly binds to GABA_B_ receptor^64^. If clozapine directly activates the GABA_B_ receptor, it could reasonably explain mechanisms of not only the effectiveness of clozapine for treatment-resistant schizophrenia but also specific features of benzodiazepine-resistance of clozapine discontinuation catatonia^64–66^. Stimulatory effects of clozapine on the astroglial release of L-BAIBA, a candidate GABAergic gliotransmitter, are sufficient to augment the GABA_B_ receptor hypothesis regarding the pathophysiology of clinical action and adverse reaction of clozapine^64,65^.

### Effects of clozapine and brexpiprazole on transmission associated with L-BAIBA

This study demonstrated that clozapine enhanced AMPK signalling, similar to previous studies^10^. Based on H1 receptor hypothesis^3,10,14^, we determined the effects of clozapine on intracellular levels of IP3, cAMP, AMP and ATP. Chronic clozapine administration decreased and increased levels of IP3 and AMP, respectively. These results reasonably support the H1 receptor hypothesis that clozapine enhances AMPK signalling due to suppressing IP3 production via the blockade of both H1 and 5-HT2A receptors. However, this hypothesis does not seem to explain the mechanisms underlying the inhibitory effects of brexpiprazole on AMPK signalling. Indeed, brexpiprazole decreased and increased intracellular levels of IP3 and AMP, respectively, resembling clozapine. However, brexpiprazole decreased cAMP levels, which is notably different effect from clozapine. Traditionally, it is well known that antipsychotics increase cAMP via dopamine D2 receptor blockade^67^; however, considering the receptor-binding profile of brexpiprazole, the agonistic effect on 5-HT1A receptor that inhibits adenylyl cyclase and inhibition of 5-HT7 receptor that activates adenylyl cyclase, rationally supports the hypothesis that brexpiprazole decreased cAMP levels^43,44,51,68^. It has been established that cAMP enhances and inhibits AMPK signalling via activation of EPAC and PKA activities, respectively^58^,whereas decreased cAMP levels by brexpiprazole seem to contribute to the suppression of AMPK signalling^43^. In contrast to the complicated and inconsistent regulatory mechanism of AMPK activity by second messengers, the hypothalamic intracellular level of L-BAIBA regulates AMPK signalling consistently. Increasing and decreasing hypothalamic intracellular levels of L-BAIBA, which positively regulates AMPK signalling, by respective clozapine and brexpiprazole suggest that L-BAIBA possibly contributes to weight gain and metabolic complications associated with antipsychotics. Therefore, clozapine and brexpiprazole increased and decreased the hypothalamic L-BAIBA level, respectively, whereas neither antipsychotics affected total BAIBA enantiomer levels in the peripheral organs. In other words, discrepant effects of antipsychotics on AMPK signalling between the peripheral organs and the brain may support the idea that L-BAIBA plays fundamental roles in regulating AMPK signalling in the hypothalamus.

It has been established that AMPK is not only a fundamental regulator of cellular metabolism but that hypothalamic AMPK signalling also plays a major role in regulating whole-body energy balance^5^. Several promising agents that activate AMPK have been identified and are being tested in clinical trials^69^; however, direct targeting of hypothalamic AMPK presents important challenges since activation of hypothalamic AMPK signalling increases food intake and exacerbates metabolic disorders^70^. Therefore, clinical treatment must selectively target the inhibition of peripheral AMPK, avoiding any possible hypothalamic effects^5^. When developing novel therapeutic agents against metabolic disorders originating from enhanced hypothalamic AMPK signalling, including those induced by antipsychotics, the present results highlighting the suppression of L-BAIBA synthesis in the hypothalamus is a potential target for drug discovery.

Chronic administration of vigabatrin (75–300 mg/kg/day for 14 d) dose-dependently decreased rat weight (up to 20% of the original body weight) by decreasing appetite^71,72^. Clinically, vigabatrin ranks as an antiepileptic drug with minimal effects on weight loss^73^. Based on these clinical findings, ABAT suppression by vigabatrin does not seem to be a promising target for reducing antipsychotic-induced weight gain and metabolic complications originating from hypothalamic AMPK signalling activation. On the other hand, mitochondrial carbonic topiramate and zonisamide have been reported to be effective against antipsychotic-induced weight gain^74^. Unfortunately, these two antiepileptic drugs are inappropriate for patients with epilepsy and comorbid psychotic symptoms and mood disorders^75^. Based on the results of this study, both hypothalamic changing L-BAIBA levels by chronic administrations of clozapine and brexpiprazole are probably not modulated by ABAT activity. Instead, their mechanism lies upstream of ABAT in the L-BAIBA metabolic pathway. Specifically, in the transamination/oxidative reaction of L-valine or transamination between L-methyl-malonyl semialdehyde and L-glutamate, we can find promising target molecules for modulating the activity of clozapine and brexpiprazole^57^.

## Conclusion

To clarify the pathophysiology of antipsychotics-induced weight gain and metabolic complications, this study determined the effects of clozapine (high risk of weight gain) and brexpiprazole (relatively low risk of weight gain) on intracellular and extracellular levels of BAIBA enantiomers, which are activators of AMPK signalling. This study identified L-BAIBA as the dominant BAIBA enantiomer in the brain and suggested it as a candidate GABAergic gliotransmitter. In the extracellular space, L-BAIBA displayed GABA_B_ receptor agonistic action, whereas L-BAIBA intracellularly indicated the possible function of the AMPK activator. Chronic administration of effective doses of clozapine and brexpiprazole increased and decreased intracellular L-BAIBA levels, respectively, in both the hypothalamus and cultured astrocytes. These results suggest that enhancing hypothalamic AMPK signalling by increasing intracellular L-BAIBA levels is, at least partially, involved in antipsychotic-induced weight gain and metabolic complications. Additional studies that determine the effects of other antipsychotics on L-BAIBA levels and AMPK signalling can clarify the detailed pathophysiology of antipsychotic-induced weight gain and metabolic complications and may contribute to the identification of novel treatment targets for antipsychotic-resistant schizophrenia.

## References

[1] Lawrence D, Hancock KJ, Kisely S (2013). The gap in life expectancy from preventable physical illness in psychiatric patients in Western Australia: retrospective analysis of population based registers. BMJ.

[2] De Hert M (2011). Physical illness in patients with severe mental disorders. I. Prevalence, impact of medications and disparities in health care. World Psychiatry.

[3] Carli M (2021). Atypical antipsychotics and metabolic syndrome: from molecular mechanisms to clinical differences. Pharmaceuticals.

[4] Wu H (2022). Antipsychotic-induced weight gain: dose-response meta-analysis of randomized controlled trials. Schizophr. Bull..

[5] Lopez M (2022). Hypothalamic AMPK as a possible target for energy balance-related diseases. Trends Pharmacol. Sci..

[6] Foretz M, Guigas B, Bertrand L, Pollak M, Viollet B (2014). Metformin: from mechanisms of action to therapies. Cell Metab..

[7] Oh KJ (2011). Atypical antipsychotic drugs perturb AMPK-dependent regulation of hepatic lipid metabolism. Am. J. Physiol. Endocrinol. Metab..

[8] Li DJ (2021). Brexpiprazole caused glycolipid metabolic disorder by inhibiting GLP1/GLP1R signaling in rats. Acta Pharmacol. Sin..

[9] Siskind DJ, Leung J, Russell AW, Wysoczanski D, Kisely S (2016). Metformin for clozapine associated Obesity: a systematic review and meta-analysis. PLoS One.

[10] Kim SF, Huang AS, Snowman AM, Teuscher C, Snyder SH (2007). From the cover: antipsychotic drug-induced weight gain mediated by histamine H1 receptor-linked activation of hypothalamic AMP-kinase. Proc. Natl Acad. Sci. USA.

[11] Lian J, Huang XF, Pai N, Deng C (2014). Betahistine ameliorates olanzapine-induced weight gain through modulation of histaminergic, NPY and AMPK pathways. Psychoneuroendocrinology.

[12] Fukuyama K, Motomura E, Okada M (2022). Brexpiprazole reduces 5-HT7 receptor function on astroglial transmission systems. Int. J. Mol. Sci..

[13] Okada M, Fukuyama K, Motomura E (2022). Dose-dependent biphasic action of quetiapine on AMPK signalling via 5-HT7 receptor: exploring pathophysiology of clinical and adverse effects of quetiapine. Int. J. Mol. Sci..

[14] He M, Deng C, Huang XF (2013). The role of hypothalamic H1 receptor antagonism in antipsychotic-induced weight gain. CNS Drugs.

[15] Okada M, Yoshida S, Zhu G, Hirose S, Kaneko S (2005). Biphasic actions of topiramate on monoamine exocytosis associated with both soluble N-ethylmaleimide-sensitive factor attachment protein receptors and Ca(2+)-induced Ca(2+)-releasing systems. Neuroscience.

[16] de Brito OM, Scorrano L (2010). An intimate liaison: spatial organization of the endoplasmic reticulum-mitochondria relationship. EMBO J..

[17] Decrock E (2013). IP3, a small molecule with a powerful message. Biochim. Biophys. Acta.

[18] Ishima T (2015). Potentiation of neurite outgrowth by brexpiprazole, a novel serotonin-dopamine activity modulator: a role for serotonin 5-HT1A and 5-HT2A receptors. Eur. Neuropsychopharmacol..

[19] Roberts LD (2014). beta-Aminoisobutyric acid induces browning of white fat and hepatic beta-oxidation and is inversely correlated with cardiometabolic risk factors. Cell Metab..

[20] Jung TW (2015). BAIBA attenuates insulin resistance and inflammation induced by palmitate or a high fat diet via an AMPK-PPARdelta-dependent pathway in mice. Diabetologia.

[21] Shi CX (2016). beta-aminoisobutyric acid attenuates hepatic endoplasmic reticulum stress and glucose/lipid metabolic disturbance in mice with type 2 diabetes. Sci. Rep..

[22] Vemula H, Kitase Y, Ayon NJ, Bonewald L, Gutheil WG (2017). Gaussian and linear deconvolution of LC-MS/MS chromatograms of the eight aminobutyric acid isomers. Anal. Biochem..

[23] Solem E, Jellum E, Eldjarn L (1974). The absolute configuration of β-aminoisobutyric acid in human serum and urine. Clin. Chim. Acta.

[24] Lee IS, Nishikimi M, Inoue M, Muragaki Y, Ooshima A (1999). Specific expression of alanine-glyoxylate aminotransferase 2 in the epithelial cells of Henle’s loop. Nephron.

[25] Pollitt RJ, Green A, Smith R (1985). Excessive excretion of beta-alanine and of 3-hydroxypropionic, R- and S-3-aminoisobutyric, R- and S-3-hydroxyisobutyric and S-2-(hydroxymethyl)butyric acids probably due to a defect in the metabolism of the corresponding malonic semialdehydes. J. Inherit. Metab. Dis..

[26] Roe CR (1998). Methylmalonic semialdehyde dehydrogenase deficiency: psychomotor delay and methylmalonic aciduria without metabolic decompensation. Mol Genet. Metab..

[27] Kakimoto Y, Kanazawa A, Taniguchi K, Sano I (1968). Beta-aminoisobutyrate-alpha-ketoglutarate transaminase in relation to beta-aminoisobutyric aciduria. Biochim. Biophys. Acta.

[28] Kupiecki FP, Coon MJ (1957). The enzymatic synthesis of beta-aminoisobutyrate, a product of valine metabolism, and of beta-alanine, a product of beta-hydroxypropionate metabolism. J. Biol. Chem..

[29] Fagerberg L (2014). Analysis of the human tissue-specific expression by genome-wide integration of transcriptomics and antibody-based proteomics. Mol. Cell Proteomics.

[30] Silverman RB (2018). Design and mechanism of GABA aminotransferase inactivators. Treatments for epilepsies and addictions. Chem. Rev..

[31] Tanahashi S (2012). Novel delta1-receptor agonist KNT-127 increases the release of dopamine and L-glutamate in the striatum, nucleus accumbens and median pre-frontal cortex. Neuropharmacology.

[32] Horikoshi T, Asanuma A, Yanagisawa K, Anzai K, Goto S (1988). Taurine and beta-alanine act on both GABA and glycine receptors in Xenopus oocyte injected with mouse brain messenger RNA. Brain Res..

[33] Schmieden V, Betz H (1995). Pharmacology of the inhibitory glycine receptor: agonist and antagonist actions of amino acids and piperidine carboxylic acid compounds. Mol. Pharmacol..

[34] Lilley E (2020). ARRIVE 2.0 and the British Journal of Pharmacology: Updated guidance for 2020. Br. J. Pharmacol..

[35] Tanahashi S, Yamamura S, Nakagawa M, Motomura E, Okada M (2012). Clozapine, but not haloperidol, enhances glial D-serine and L-glutamate release in rat frontal cortex and primary cultured astrocytes. Br. J. Pharmacol..

[36] O’Connor WT, O’Shea SD (2015). Clozapine and GABA transmission in schizophrenia disease models: establishing principles to guide treatments. Pharmacol. Ther..

[37] Fukuyama K, Okada M (2021). Effects of atypical antipsychotics, clozapine, quetiapine and brexpiprazole on astroglial transmission associated with Connexin43. Int. J. Mol. Sci..

[38] Dewey SL (1999). A pharmacologic strategy for the treatment of nicotine addiction. Synapse.

[39] Fukuyama K, Okubo R, Murata M, Shiroyama T, Okada M (2020). Activation of astroglial connexin is involved in concentration-dependent double-edged sword clinical action of clozapine. Cells.

[40] Lucke A (1998). Gabapentin potentiation of the antiepileptic efficacy of vigabatrin in an in vitro model of epilepsy. Br. J. Pharmacol..

[41] Balla A (2009). GABAB/NMDA receptor interaction in the regulation of extracellular dopamine levels in rodent prefrontal cortex and striatum. Neuropharmacology.

[42] Yamamura S (2013). ONO-2506 inhibits spike-wave discharges in a genetic animal model without affecting traditional convulsive tests via gliotransmission regulation. Br. J. Pharmacol..

[43] Fukuyama K, Motomura E, Shiroyama T, Okada M (2022). Impact of 5-HT7 receptor inverse agonism of lurasidone on monoaminergic tripartite synaptic transmission and pathophysiology of lower risk of weight gain. Biomed. Pharmacother..

[44] Okada M, Fukuyama K, Ueda Y (2019). Lurasidone inhibits NMDA receptor antagonist-induced functional abnormality of thalamocortical glutamatergic transmission via 5-HT7 receptor blockade. Br. J. Pharmacol..

[45] Fukuyama K, Okada M (2022). High frequency oscillations play important roles in development of epileptogenesis/ictogenesis via activation of astroglial signallings. Biomed. Pharmacother..

[46] Latchoumane CV, Ngo HV, Born J, Shin HS (2017). Thalamic spindles promote memory formation during sleep through triple phase-locking of cortical, thalamic, and hippocampal rhythms. Neuron.

[47] Iversen LL, Glowinski J (1966). Regional studies of catecholamines in the rat brain. II. Rate of turnover of catecholamines in various brain regions. J. Neurochem..

[48] Azevedo C, Saiardi A (2006). Extraction and analysis of soluble inositol polyphosphates from yeast. Nat. Protoc..

[49] Shiroyama T, Fukuyama K, Okada M (2021). Distinct effects of escitalopram and vortioxetine on astroglial L-glutamate release associated with connexin43. Int. J. Mol. Sci..

[50] Fukuyama K, Fukuzawa M, Shiroyama T, Okada M (2020). Pathogenesis and pathophysiology of autosomal dominant sleep-related hypermotor epilepsy with S284L-mutant alpha4 subunit of nicotinic ACh receptor. Br. J. Pharmacol..

[51] Okada M, Fukuyama K, Okubo R, Shiroyama T, Ueda Y (2019). Lurasidone sub-chronically activates serotonergic transmission via desensitization of 5-HT1A and 5-HT7 receptors in dorsal raphe nucleus. Pharmaceuticals.

[52] Fukuyama K, Kato R, Murata M, Shiroyama T, Okada M (2019). Clozapine normalizes a glutamatergic transmission abnormality induced by an impaired NMDA receptor in the thalamocortical pathway via the activation of a group III metabotropic glutamate receptor. Biomolecules.

[53] Curtis MJ (2018). Experimental design and analysis and their reporting II: updated and simplified guidance for authors and peer reviewers. Br. J. Pharmacol..

[54] Armstrong MD, Yates K, Kakimoto Y, Taniguchi K, Kappe T (1963). Excretion of β-aminoisobutyric acid by man. J. Biol. Chem..

[55] Gejyo F, Kinoshita Y, Ikenaka T (1976). Identification of beta-aminoisobutyric acid in uremic serum. Clin Chim Acta.

[56] Stautemas J (2019). Acute aerobic exercise leads to increased plasma levels of R- and S-beta-aminoisobutyric acid in humans. Front. Physiol..

[57] Tanianskii DA, Jarzebska N, Birkenfeld AL, O’Sullivan JF, Rodionov RN (2019). Beta-aminoisobutyric acid as a novel regulator of carbohydrate and lipid metabolism. Nutrients.

[58] Aslam M, Ladilov Y (2022). Emerging role of cAMP/AMPK signaling. Cells.

[59] Kowalchuk C, Kanagasundaram P, Belsham DD, Hahn MK (2019). Antipsychotics differentially regulate insulin, energy sensing, and inflammation pathways in hypothalamic rat neurons. Psychoneuroendocrinology.

[60] Himmerich H, Minkwitz J, Kirkby KC (2015). Weight gain and metabolic changes during treatment with antipsychotics and antidepressants. Endocr. Metab. Immune Disord. Drug Targets.

[61] Yoshida S, Okada M, Zhu G, Kaneko S (2007). Carbamazepine prevents breakdown of neurotransmitter release induced by hyperactivation of ryanodine receptor. Neuropharmacology.

[62] Yoshida S (2010). Effects of valproate on neurotransmission associated with ryanodine receptors. Neurosci. Res..

[63] Okada M, Zhu G, Yoshida S, Hirose S, Kaneko S (2004). Protein kinase associated with gating and closing transmission mechanisms in temporoammonic pathway. Neuropharmacology.

[64] Nair PC, McKinnon RA, Miners JO, Bastiampillai T (2020). Binding of clozapine to the GABAB receptor: clinical and structural insights. Mol. Psychiatry.

[65] Hirjak D, Northoff G, Taylor SF, Wolf RC (2021). GABAB receptor, clozapine, and catatonia-a complex triad. Mol. Psychiatry.

[66] Lander M, Bastiampillai T, Sareen J (2018). Review of withdrawal catatonia: what does this reveal about clozapine. Transl. Psychiatry.

[67] Vanhauwe JF, Ercken M, van de Wiel D, Jurzak M, Leysen JE (2000). Effects of recent and reference antipsychotic agents at human dopamine D2 and D3 receptor signaling in Chinese hamster ovary cells. Psychopharmacology.

[68] Okada M, Matsumoto R, Yamamoto Y, Fukuyama K (2021). Effects of subchronic administrations of vortioxetine, lurasidone, and escitalopram on thalamocortical glutamatergic transmission associated with serotonin 5-HT7 receptor. Int. J. Mol. Sci..

[69] Steinberg GR, Carling D (2019). AMP-activated protein kinase: the current landscape for drug development. Nat. Rev. Drug Discov..

[70] Okamoto S (2018). Activation of AMPK-regulated CRH neurons in the PVH is sufficient and necessary to induce dietary preference for carbohydrate over fat. Cell Rep..

[71] DeMarco A (2008). Subchronic racemic gamma vinyl-GABA produces weight loss in Sprague Dawley and Zucker fatty rats. Synapse.

[72] Gibson JP (1990). Chronic toxicity studies with vigabatrin, a GABA-transaminase inhibitor. Toxicol. Pathol..

[73] Buraniqi E, Dabaja H, Wirrell EC (2022). Impact of antiseizure medications on appetite and weight in children. Paediatr. Drugs.

[74] Supuran CT (2022). Anti-obesity carbonic anhydrase inhibitors: challenges and opportunities. J. Enzyme Inhib. Med. Chem..

[75] Villanueva V (2021). Initiating antiepilepsy treatment: an update of expert consensus in Spain. Epilepsy Behav..

